# Influence of maternal age on the birthweight of infants delivered from frozen-thawed blastocyst transfer cycles

**DOI:** 10.3389/fendo.2023.1195256

**Published:** 2023-07-10

**Authors:** Xue Wang, YaLing Xiao, Tao Tao, Wei Xiong

**Affiliations:** Department of Gynecology Endocrine and Reproductive Center, State Key Laboratory of Complex Severe and Rare Disease, Peking Union Medical College Hospital, Chinese Academy of Medical Sciences and Peking Union Medical College, Beijing, China

**Keywords:** maternal age, birthweight, frozen-thawed blastocyst transfer, low birth weight, premature birth

## Abstract

The aim of this study was to investigate whether maternal age had an effect on the birthweight of singletons delivered from frozen-thawed blastocyst transfer (FBT) cycles. A total of 1203 FBT cycles occurring between July 2011 and June 2021 at a single centre were retrospectively analysed. Based on the maternal age at FBT, the patients were divided into four groups: <30, 30–34, 35−37, and ≥38 years of age. Main outcomes measured included singleton birthweights, preterm births, large-for-gestational-age (LGA) and small-for-gestational-age (SGA) live births among the groups. There was no significant difference in birth weight among the four groups, while the highest birth weight was found in the <30 years group. The incidence of very preterm births and very low birth weights demonstrated an increasing trend with age; on the contrary, the incidence of preterm births, low birth weight (LBW), high birth weight and LGA and SGA live births gradually decreased with increasing age, but these differences were not statistically significant among groups (P>0.05, respectively). Although the proportion of females was lower than that of males, the difference was not statistically significant among the groups. After adjusting for possible confounders, maternal age was found to have no effect on adverse neonatal outcomes in the regression analyses (P>0.05). Birthweight in singleton births from FBT was not affected by maternal age.

## 1 Introduction

With the development of assisted reproductive technology (ART), more women seek ART treatment due to infertility, and consequently more children are delivered through ART (1). In recent years, many women choose to delay childbearing due to various reasons, with most of them over 40 years of age (2). As is well known, age is a crucial factor affecting fertility (3) and the quality and quantity of oocytes decrease significantly as women age, resulting in diminished fertility (4). In addition, the incidence of adverse birth outcomes increases with increasing maternal age (5, 6). Women with advanced age are more likely than younger women to develop pregnancy-related diseases such as gestational hypertension, gestational diabetes mellitus (GDM), and perinatal disorders that particularly inlcude age-related perinatal mortality, PTB, and LBW (7). Several studies have reported that maternal age has negative effects on neonatal outcomes in natural pregnancies (8–10). However, it is unclear whether these conclusions can be applied to women undergoing ART. Theoretically, older age combined with ART may be more likely to have poor birth outcomes such as LBW or PTB (11–13). Thus, it is crucial to investigate whether maternal age has an effect on birth outcomes in ART cycles.

Currently, literature on the association between the birthweight of newborns and maternal age in IVF cycles is limited. Previously published literature has focused on fresh transfer cycles (8, 9, 14). However, confounding factors must be considered in fresh cycles as the patients are in a high oestrogen environment due to large amounts of gonadotropins used for ovulation stimulation. Some studies suggest that high oestrogen levels may lead to histological changes in the endometrium and could influence gene expression, which could affect embryo implantation and placental development, resulting in complications such as LBW (15–18). Analysis of neonatal outcomes from frozen embryo transfer (FET) cycles seems to be an alternative option because FET does not require high oestrogen levels to stimulate ovulation, keeping the endometrium conducive to early embryo development and implantation (19). Two recent studies analysed whether maternal age had an effect on neonatal birth weight from FET cycles and found that maternal age did not affect birth weight (20, 21). However, embryo quality, which may affect neonatal outcomes, was not considered in the study by Ni et al. (20), and the majority of the transferred embryos were cleavage embryos in these two studies, with low proportions of blastocyst transfer (20, 21). Some studies have suggested that neonatal outcomes are different between frozen cleavage embryos transfer (FCET) and FBT, and that FBT has better outcomes than FCET (22). However, to date, there has been no literature analysing the impact of maternal age on the birth weight of neonates conceived from FBT cycles. Therefore, the present study aims to investigate whether maternal age affects the birth weight of singletons derived from FBT cycles.

## 2 Materials and methods

### 2.1 Study population and patient grouping

This study retrospectively analysed data from patients who underwent FBT at the Gynecological Endocrinology and Assisted Reproduction Center of Peking Union Medical College Hospital between July 2011 to June 2021. The inclusion criteria were as follows: (1) singleton birth and (2) women with one or two frozen-thawed blastocyst transfers. The exclusion criteria were as follows: (1) disappearance of one twin after the third trimester of pregnancy; (2) congenital uterine malformations; (3) history of cancer such as ovarian; (4) medical diseases such as thyroid dysfunction, diabetes, liver disease, and hypertension; (5) pregnancy related disease such as gestational hypertension, preeclampsia, and GDM; (6) chromosomal abnormalities in either the father or mother; (7) oocytes derived from an ovum donation cycle; (8) sperm derived from epididymal puncture or testicular puncture, and (9) preimplantation genetic test cycle. Data from 1203 patients were included. According to maternal age at FBT, the patients were grouped into four groups: <30, 30–34, 35−37, and ≥38 years of age. The requirement for patient consent was waived owing to the retrospective nature of the study. This study was approved by the Ethics Committee of the Peking Union Medical College Hospital.

### 2.2 Treatment protocol and blastocyst vitrification

In our centre, two ovulation stimulation regimens are routinely utilized that include gonadotropin-releasing hormone (GnRH) agonist or GnRH antagonist protocols. When more than three follicles were 18 mm in diameter, human chorionic gonadotropin (HCG) was then injected to induce final oocyte maturation. Transvaginal ultrasound oocyte retrieval was performed 38 h following trigger administration. On the day of oocyte retrieval, insemination was performed on the basis of paternal semen quality. On the third day after fertilization, one or two good cleavage embryos were chosen for transfer. All other embryos were continuously cultured to the blastocyst stage with the informed consent of the patients. If blastocysts formed on day 5 (D5) or 6 (D6), they were scored morphologically and cryopreserved. We referred to the Gardner scoring system for blastocyst evaluation criteria (23). The quality of the inner cell mass (ICM) and trophoblast mass (TE) were divided into grades A, B, and C, respectively. If the ICM was grade A or B, the blastocysts could be selected for cryopreservation. In contrast, if the ICM was Grade C, it would be abandoned. Blastocysts with ICM grades A or B and TE grades A or B were defined as good quality embryos. The selected blastocysts were artificially shrunk using a laser system before being frozen and vitrified with dimethyl sulfoxide, ethylene glycol, sucrose, and Ficoll as cryoprotectants, and cryopreserved in liquid nitrogen.

### 2.3 Warming of blastocyst and endometrial preparation

The blastocysts were thawed on the morning of FBT using a sequential dilution solution of sucrose, hatched by laser, and cultured for approximately 2 h until embryo transfer. Endometrial preparation was performed in two different ways. The natural cycle method was used for patients who had regular menstrual cycles with normal ovulation, and HCG was injected for triggering ovulation. In the artificial cycle method, oestradiol valerate (Progynova, Schering, Germany) was administered orally on days 2–4 of menstruation, and different starting doses (2–18mg/day) were given for the women on basis of their previous endometrial conditions. When the thickness of the endometrium reached 8 mm, progesterone (20mg×2d, 40mg×2d,20-40mg×1d) was injected intramuscularly for 5 days. On the 6th day, the blastocysts were thawed and transferred. Progesterone vaginal gel 8% (Crinone, Merck Serono, Feltham, UK) or intramuscularly ingested progesterone were used for luteal support.

### 2.4 Outcome parameters

PTB was defined as delivery occurring at a gestational age ≥28 weeks and <37 weeks. Very PTB referred to gestational age at birth <32 weeks. LBW referred to weight at birth <2500 g and VLBW referred to weight at birth <1500 g, while macrosomia or high birth weight (HBW) was defined as weight at birth ≥4,000 g. SGA infants were infants whose birth weight were below the 10th percentile of the average weight of infants of the same gestational age. LGA infants referred to newborns whose birth weight were above the 90th percentile of the average weight of infants of the same gestational age.

### 2.5 Statistical analysis

Statistical analyses were performed using SPSS software (Statistical Package for the Social Sciences, version 22.0 IBM, Armonk, NY, USA). The comparison of means between groups depended on data distribution. If the data was continuous, it was analysed by one-way analysis of variance (one-way ANOVA). If the data was categorical, it was analysed by chi-square test or Fisher’s exact test. Bonferroni correction was used to adjust for multiple comparisons. A regression model was used to analyse the influence of maternal age on the birth outcomes of newborns. The <30 years old group was designated as the reference group. Results with P<0.05 were considered statistically significant.

## 3 Results

Baseline characteristics are presented in **Table 1**. The proportions of all five groups (<30, 30−34, 35−37, and ≥38 years) were 15.5%, 45.1%, 23.3%, and 16.1%, respectively. Comparisons among the groups showed significant statistical differences in the years of infertility duration, age at oocyte retrieval, infertility causes, types, and parity. Younger patients had male infertility factors as the primary cause of infertility than did older women (46.2%, 33.3%, 35.0%, and 36.6%, P=0.04). Significant differences in the endometrial preparation protocol, number of transferred embryos, insemination method of blastocyst-derived embryos, and good quality embryos transferred were observed among groups (P<0.01, respectively). With increasing age, the rate of good embryo transfers also decreased (76.4%, 72.1%, 65.2%, and 65.0%, P<0.01).

**Table 1:** Baseline characteristics of the five study groups.

Characteristic	<30	30-34	35-37	≥38	P
**Patients (n)**	186	543	280	194	
**Mean age**	27.6 ± 2.0	32.6 ± 1.4	36.3 ± 0.9	39.9 ± 4.7	<0.01
**Age in oocyte**	27.0 ± 2.2	31.6 ± 2.3	35.0 ± 1.7	38.1 ± 2.6	<0.01
**Duration of sterility**	3.6 ± 2.0	4.6 ± 2.5	5.6 ± 3.0	6.8 ± 3.7	<0.01
**BMI**	20.9 ± 2.1	21.0 ± 2.1	20.9 ± 2.0	21.1 ± 2.0	0.35
Infertility causes					0.02
Tubal factors	41(22.0)	134(24.7)	86(30.7)	60(30.9)	0.12
Endometriosis	14(7.5)	91 (16.8)	58(20.7)	36(18.6)	<0.01
Abnormal ovulation	43(23.1)	129(23.8)	68(24.3)	35(18.0)	0.43
Male factors	86(46.2)	181(33.3)	98(35.0)	71(36.6)	0.04
Unexplained	9(4.8)	15(2.8)	6(2.1)	9(4.6)	0.26
Parity					<0.01
0	180(96.8)	522(96.1)	248(88.6)	137(70.6)	
≥1	6(3.2)	21(3.9)	32(11.4)	57(29.4)	
Type of infertility					<0.01
Primary	123(66.1)	344(63.4)	137(48.9)	77(39.7)	
Secondary	63(33.9)	199(36.6)	143(51.1)	117(60.3)	
Ovulation stimulation regimen					0.06
GnRHa	114(61.3)	327(60.2)	154(55.0)	98(50.5)	
GnRHan	72(38.7)	216(39.8)	126(45.0)	96(49.5)	
Endometrial preparation protocol					<0.01
Natural cycle	32(17.2)	137(25.2)	73(26.1)	71(36.6)	
Artificial cycle	154(82.8)	406(74.8)	207(73.9)	123(63.4)	
Fertilization method					0.02
IVF	104(55.9)	360(66.3)	196(70.0)	133(68.6)	
ICSI	82(44.1)	183(33.7)	84(30.0)	61(31.4)	
Number of blastocysts transferred					0.02
1	67(36.0)	155(28.5)	106(37.9)	56(28.9)	
2	119(64.0)	388(71.5)	174(62.1)	138(71.1)	
**Endometrial thickness (mm)**	10.1 ± 1.5	10.2 ± 1.7	9.9 ± 1.4	10.1 ± 1.5	0.21
**Rate of good blastocyst transferred (%)**	233(76.4)	672(72.1)	296(65.2)	217(65.0)	<0.01
Blastocyst development speed (%)					0.13
D5	191(62.6)	562(60.4)	259(57.0)	179(53.9)	
D6	114(37.4)	369(39.6)	195(43.0)	153(41.1)	

IVF, *in vitro* fertilization; BMI, body mass index; ICSI, intracytoplasmic sperm injection; D5, Day 5; D6, Day 6; Results with P<0.05 were considered statistically signiﬁcant.

Comparisons of neonatal outcomes among the four groups are shown in **Table 2**. Most patients selected caesarean section as the method of delivery (67.2%, 62.8%, 66.4%, and 76.8%; P=0.01). Birth weight, body length, and gestational age were similar among groups; however, birth weight was highest in the <30 years group. Interestingly, the prevalence of LBW, HBW and PTB showed a declining trend with age, and the women who were ≥38 years had the lowest incidence of LBW, HBW, and PTB; conversely, the incidence of VLBW and very PTB (VPTB) showed an increasing trend with age, but the differences had no statistically significance (P>0.05 respectively). Moreover, no significant difference in LGA or SGA was observed among groups (P>0.05 respectively). The overall ratio of males/females was 1.1 in this study. The <30 years group had the highest proportion of males (57.0%), whereas the 35-37 years group had the lowest (47.5%) proportion, although the difference had no statistical significance (P>0.05).

**Table 2:** Comparisons of neonatal outcomes among the five study groups.

Patients (n)	<30	30-34	35-37	≥38	P
	186	543	280	194	
Mode of delivery					
Vaginal	61 (32.8)	202 (37.2)	94 (33.6)	45 (23.2)	0.01
Caesarean section	125 (67.2)	341 (62.8)	186 (66.4)	149 (76.8)	
Length at birth (cm)	49.8 ± 2.5	49.7 ± 2.7	49.8 ± 2.3	49.6 ± 2.6	0.73
Birthweight (g)	3317.6 ± 570.2	3297.1 ± 550.0	3281.9 ± 559.9	3293.0 ± 505.0	0.93
Birthweight categories					
LBW (<2500 g)	13 (7.0)	35 (6.4)	21 (7.5)	10 (5.2)	0.78
VLBW (<1500 g)	1 (0.5)	1 (0.2)	3 (1.1)	2 (1.0)	0.34
HBW ≥4000 g	15 (8.1)	49 (9.0)	23 (8.2)	10 (5.2)	0.41
Gestational age (d)	271.2 ± 13.9	272.1 ± 13.3	271.4 ± 14.1	270.0 ± 12.9	0.30
PTB(<37w)	21 (11.3)	55 (10.1)	30 (10.7)	16 (8.2)	0.77
VPTB (<32w)	2 (1.1)	7 (1.3)	4 (1.4)	4 (2.1)	0.85
Birthweight percentiles					
LGA (>90^th^ percentile)	42 (22.6)	117 (21.5)	49 (17.5)	33 (17.0)	0.29
SGA (<10^th^ percentile)	12 (6.5)	36 (6.6)	16 (5.7)	6 (3.1)	0.33
Newborn’s gender					0.20
Male	106 (57.0)	291 (53.6)	133 (47.5)	103 (53.1)	
Female	80 (43.0)	252 (46.4)	147 (52.5)	91 (46.9)	

LBW, low birth weight; VLBW, very low birth weight; HBW, high birth weight; PTB, preterm birth; VPTB, very preterm birth; LGA, large for gestational age; SGA, small for gestational age; Results with P<0.05 were considered statistically signiﬁcant.

Logistic regression analysis of adverse neonatal outcomes, with mothers <30 years of age as the reference group, revealed that the incidence of PTB, VPTB, LBW, VLBW, SGA, and LGA were similar among the five groups, as presented in **Table 3**. Even after adjusting for confounding factors that could affect neonatal outcomes including maternal BMI, age at oocyte retrieval, duration of infertility, causes of infertility, fertilization *via* intracytoplasmic sperm injection (ICSI), protocols for endometrial preparation, endometrial thickness, quality and quantity of blastocysts transferred, speed of blastocyst development, and sex ratio, there was still no significant statistical difference. Although VLBW and VPTB were also analysed, their incidence was too low, and the data was too sparse to be meaningful.

**Table 3:** Crude and adjusted odds ratios (95%confidence intervals) for neonatal outcomes in singleton births at selected maternal ages.

	30-34		35-37		≥38		P	Adjusted P
	RR (95% CI)	Adjusted RR (95% CI)	RR (95% CI)	Adjusted RR (95% CI)	RR (95% CI)	Adjusted RR (95% CI)		
LBW	0.92(0.47-1.77)	0.59(0.16-2.12)	1.08(0.53-2.21)	0.96(0.16-5.81)	0.72(0.31-1.69)	0.80(0.07-9.15)	0.78	0.59
VLBW	1.37(0.15-12.36)	1.10(0.04-29.17)	1.33(0.12-14.78)	2.59(0.02-297.22)	1.93(0.17-21.43)	NA	0.96	0.90
HBW	1.13(0.62-2.07)	2.36(0.65-8.63)	1.02(0.52-2.01)	2.86(0.50-16.37)	0.62(0.27-1.42)	1.96(0.20-19.59)	0.42	0.38
PTB	0.89(0.52-1.51)	0.81(0.28-2.36)	0.94(0.52-1.70)	1.30(0.29-5.88)	0.71(0.36-1.40)	1.59 (0.22-11.75)	0.77	0.58
VPTB	1.20(0.25-5.84)	0.59(0.05-7.86)	1.33(0.24-7.35)	2.06(0.05-82.25)	1.94(0.35-10.70)	1.27(0.01-200.53)	0.85	0.60
SGA	1.03(0.52-2.02)	1.02(0.27-3.88)	0.88(0.41-1.90)	0.50(0.07-3.74)	0.46(0.17-1.26)	0.51(0.03-7.56)	0.35	0.65
LGA	0.94(0.63-1.41)	0.85(0.39-1.89)	0.96(0.61-1.49)	0.93(0.30-2.82)	0.70(0.42-1.17)	0.62(0.14-2.80)	0.51	0.75

LBW, low birth weight; VLBW, very low birth weight; HBW, high birth weight; PTB, preterm birth; VPTB, very preterm birth; LGA, large for gestational age; SGA, small for gestational age; OR, odds ratio; CI, confidence interval; Results with P<0.05, were considered statistically signiﬁcant.

## 4 Discussion

In our study, there were no significant differences in the prevalence of LBW, PTB, SGA, or LGA among the four groups, and the birth weight of singletons derived from FBT did not change with increasing maternal age, similar to previous studies (8, 9, 14, 20, 21, 24). Wennberg et al. analysed the effect of maternal age on neonatal outcomes conceived *via* ART and natural pregnancies. They found that the incidence of PTB, LBW and SGA increased with maternal age in the spontaneous conception group, whereas adverse neonatal outcomes as mentioned did not increase with increasing maternal age in the ART group (9). Moaddab et al., found that neonatal outcomes for women <40 years and >40 years did not have significant differences (14). However, the patients were grouped as either <40 years or >40 years, which was inadequate as it led to masking of valuable information. Moreover, women over 40 years of age were more likely to use oocyte donation due to decreased fertility while they did not clarify whether the patient’s embryo was their own or sourced from oocyte donation, whether the embryos were derived from fresh or FET cycles, or whether prenatal genetic screening was performed (14). Barbuscia et al. also reached a similar conclusion: the birth weight of children born through assisted reproduction did not change with an increase in maternal age up to 40 years, but the risk of LBW increased in those >40 years (6%, 95% CI:0.2, 12) (8). Recently, Ni et al. published a study on FET cycles in 12565 women which showed that maternal age had no effect on a singleton’s birth weight or newborn outcomes including PTB, LBW, and SGA, similar to our findings (20). However, the embryo quality was unknown, which could have affected neonatal outcomes, and more than 80% of the embryos transferred in the study were cleavage embryos. Birth outcomes from blastocyst transfers may differ from that of embryo cleavage transfers. A recent meta-analysis found that LGA and PTB were more likely to occur in singletons obtained through fresh and frozen blastocysts transfers than in those obtained from cleavage embryo transfers (25). Therefore, it is essential to investigate the influence of maternal age on birth outcomes of live births from FBT cycles.

As far as we know, this is the first study to investigate whether singleton birth weight after FBT is affected by maternal age. In contrast to previous studies, we recorded the number, quality, and developmental speed of the transferred embryos in detail to avoid confounding factors. As maternal age increased, the proportion of women who transferred one blastocyst decreased, and the rate of good embryo transfer also decreased. Since age is a crucial factor that can affect the clinical outcomes of ART, both the oocyte quantity and quality decline with an increase in maternal age (26, 27). Compared to younger women, those with advanced age had lower numbers of mitochondrial DNA copies in their oocytes (26) and had higher risks of aneuploidy, which led to a decline in embryo quality (27). Therefore, more high-quality embryos were transferred to younger patients in FBT cycles in this study. Although the embryo quality transferred was significantly different (P<0.01), this had no effect on singleton birth weight. The reasons for this are multifactorial and may be related to the population characteristics of women with advanced age (9).

Firstly, women of different ages seek ART treatment for different reasons, and those who are young seek ART treatment may have more health problems (8). In this study, we found that infertility in young patients was caused more by ovulation disorders or male factors, and the incidence of endometriosis was relatively higher. Among the causes of infertility in advanced age patients, tubal factors were more common, while male factors were lower than that in younger patients. Consequently, younger patients were more likely to undergo ICSI rather than IVF for fertilization. Compared with younger patients, the proportion of secondary infertility in older patients was higher. These selection biases may have affected our results and led to increased adverse neonatal outcomes in younger patients. However, in regression analysis, after these confounding factors were adjusted, age still had no effect on birth weight.

Secondly, the women with advanced age treated with ART may have better economic statuses, good exercise habits, healthier lifestyles, and better physical fitness than the younger patients; therefore, they may represent a healthier group. In addition, women with advanced age who undergo ART are more likely to experience age-related infertility, and knowing this they may pay more attention to their health during pregnancy than younger women and actively seek medical help (8, 9, 20). Even with adjustments for confounding factors (such as the number, quality, and developmental speed of transferred embryos), our study found that the maternal age still had no effect on birth weight. Therefore, even if the pregnancy rate in women with advanced age is low, infant birth weight is not affected by the mother’s age once they are pregnant through ART.

Additionally, an interesting phenomenon was observed in this study: the birth weight was lowest in the 35-37 years group; the percentage of male infants was also the lowest in this group, although there were no statistical differences. This raises the question of whether the neonatal sex ratio affects birth weight. Some studies had shown that the incidence of LGA was lower in female infants, and male infants were heavier than female infants (28–31). Kaartinen et al. (28), found that male infants were 242.7 g heavier than female infants from FBT cycles (P<0.01). A recent study found that after adjusting for confounding factors, male infants were significantly heavier than female infants (3418 g vs 3263 g), and the risk of LGA in male infants increased significantly (OR=2.8, P<0.001) (29). In this study, although the male/female sex ratio in the group of women aged 35−37 years was relatively low, the risk of LGA in this group was similar to the other groups. When multivariate analysis was performed, despite adjusting for confounding factors of sex ratio, maternal age at FET still had no effect on the birth weight of newborns. Therefore, whether sex ratio affects the relationship between birth weight and maternal age requires further investigation.

However, there were some limitations in our study. First, due to its retrospective nature of the study, some background information of patients such as their education level and economic conditions could not be obtained. Second, data such as paternal age and BMI could have affected the birth weight of newborns, but these data were missing. Third, the sample size in this study was not large enough, so a large prospective multicentre study could be considered in the future. Nevertheless, we have endeavoured to ensure the accuracy and reliability of the data in this study, and problems encountered in previous studies such as ambiguous ART methods and unclear embryo quality were avoided. We analysed the influence of the number, quality, and developmental speed of blastocysts transferred in detail and reduced the influence of embryo factors. Moreover, we followed a strict selection criterion to reduce the heterogeneity of the subjects. This study carried out the transfer of frozen-thawed blastocysts because we had more experience in blastocyst culture and vitrification. Since 2005, blastocyst culture and vitrification cryopreservation have been carried out at our centre, and the survival rate after resuscitation has reached more than 99% (32). Moreover, embryo culture conditions were stable, and the methods of embryo freezing and resuscitation remained unchanged during the research period, ensuring standardization throughout the study.

## 5 Conclusion

Birth outcomes of singletons derived from FBT cycles are not affected by maternal age. This is of great significance to older patients and clinicians as it enhances patient confidence in ART procedures, such as FBT. However, attention should be paid to the clinical application of these findings because this study only included women who delivered live births through FBT and excluded patients with pregnancy-related complications. Therefore, the influence of maternal age on the weight at birth of infants delivered from FBT still needs to be investigated in future prospective studies.

## Data availability statement

The original contributions presented in the study are included in the article/supplementary material. Further inquiries can be directed to the corresponding author.

## Ethics statement

The studies involving human participants were reviewed and approved by the Ethics Committee of the Peking Union Medical College Hospital. Written informed consent for participation was not required for this study in accordance with the national legislation and the institutional requirements.

## Author contributions

XW contributed to the conception and design of the study. XW and YX performed the literature search. TT and WX were involved in statistical analysis. XW, YX, and WX contributed to the interpretation of the results. XW was responsible for manuscript drafting. All authors contributed to the article and approved the submitted version.
